# Connected Carabids: Network Interactions and Their Impact on Biocontrol by Carabid Beetles

**DOI:** 10.1093/biosci/biaa039

**Published:** 2020-05-06

**Authors:** Stefanie E De Heij, Christian J Willenborg

**Affiliations:** Department of Agriculture at the University of Saskatchewan, Saskatoon, Saskatchewan, Canada

**Keywords:** carabids, biocontrol, interaction network, community ecology, sustainable agriculture

## Abstract

Carabid beetles can greatly contribute to biocontrol in agroecosystems, reducing both insect pests and weed seeds. However, insect foraging and feeding behavior can be highly dependent on the interaction network and spatial structure of the environment, which can make their biocontrol contributions variable. In the present article, we explore how the interaction network of carabids can affect their behavior and how spatial vegetation structure and specific agronomy practices can, in turn, affect the strength of interactions in their network. We suggest that research on carabid biocontrol should move toward an approach in which the network of interactions among pests, carabids, and other organisms within its spatial structure is evaluated, with equal focus on direct and indirect interactions, and provide examples of tools to do so. Overall, we believe this approach will improve our knowledge of carabid networks, help to elucidate the underlying mechanisms of biocontrol, and lay the foundation for future biocontrol strategies.

Carabids (Coleoptera: Carabidae) are a widespread and speciose group of beetles that have long been identified as important biocontrol agents in agricultural habitats (Lövei and Sunderland 1996). Carabids have been recognized as generalist predators that can aid in reducing the abundance of a range of insect pests and weed seeds (Winder et al. 2001, Symondson et al. 2002a, Bohan et al. 2011a, Kulkarni et al. 2015). This natural pest suppression could be enhanced by creating favorable habitats for natural enemies in agricultural landscapes, also known as conservation biocontrol (Landis et al. 2000). Diverse drivers of carabid abundance and diversity in different habitats have been recognized (Menalled et al. 2007, Blubaugh et al. 2016), and these factors can be taken into consideration in conservation biocontrol efforts aimed at pest and weed reduction by carabids. An example of such an effort is the establishment of beetle banks, which have been associated with the reduction of cereal aphids (Collins et al. 2002).

The results of biocontrol experiments with carabids for the reduction of pest insects and weed seeds are, however, inconsistent. Carabids have been shown to be beneficial agents in the reduction of certain pests but disruptive in other cases. Furthermore, the contribution of carabids to weed seed loss is often found to be variable, and the relation between granivorous carabids and weed seed loss is not always apparent. Although carabids are a comparatively well studied insect group, certain aspects of carabid life are relatively understudied—for example, how consumptive and nonconsumptive interactions with other animals shape carabid communities, feeding, and foraging behavior. Furthermore, it is not clear how the spatial structure of the vegetation and agronomic practices influence these interactions and how these features affect their potential role as biocontrol agents.

Our goal in the present article is to advocate for the incorporation of a broader ecological framework into carabid biocontrol research and to highlight those topics that are currently understudied. Incorporation of the species interaction network could help in conservation efforts (Tylianakis et al. 2010), and, similarly, it could help in the understanding, prediction, and design of carabid biocontrol efforts. We have written this text with biocontrol by carabids in mind, although the concepts we discuss could be applied to the wider context of biocontrol by arthropods. The term biocontrol is a broad concept that we use in the present article to describe the natural reduction of pests and weed seeds by arthropods, efforts to enhance that reduction, and the study of it. The definitions of terms as they are used in this synthesis can be found in box 1. We have tried to include carabid examples where they were available, although for certain topics, carabid examples are limited. This emphasizes the point that there remains much to be learned about these insects.

**Box 1. Terms and definitions.**

The definition of certain terms as used in the present article can be either narrower or broader than elsewhere. Therefore, we have defined the most important terms below as we use them within the present article.

**Biocontrol.** The reduction of unwanted species (weeds or pest insects) or their damage by arthropods. This reduction can be either directly (via consumption) or indirect (via induced behavioral changes).

**Interaction network.** The collective of species that co-occur in time and space that interact in such a way that their presence can influence the occurrence (Tylianakis et al. 2010) or behavior of another species.

**Spatial structure.** The three-dimensional space in which species interact. The spatial structure in and around crop fields is determent by factors such as crop phenotype and density, weed density and diversity, and the amount of crop residue.

**Nonconsumptive effects.** All nonlethal effects predators can have on prey, including physiological, morphological, developmental, and behavioral changes (Sheriff and Thaler 2014). In the present article, we focus on the latter.

**Alternative food.** Food other than the organism's preferred food or food other than the main food that is offered in a consumption study or that is the main focus of a field study.

## The contribution of carabids to biocontrol

Generalist insect predators, such as carabids, can play an important role in lowering insect pest populations. In agricultural fields, carabids consume a variety of prey items, such as aphids (Winder et al. 2001), slugs (Symondson et al. 2002a), and Lepidoptera larvae (Clark et al. 1994, Suenaga and Hamamura 1998). The generalist predator Pterostichus melanarius Illiger and its slug prey (Mollusca: Gastropoda) have even been found to show predator–prey oscillations similar to mammalian examples (Symondson et al. 2002a). Similarly, Winder and colleagues (2001) found a spatial coupling between P. melanarius and aphid densities (Hemiptera: Aphididae). In contrast, Firlej and colleagues (2012) did not find a coupling between carabid activity density (more than 75% P. melanarius) and soybean aphids. Strong carabid–prey relations can be the result of locally abundant prey, to which carabids react with a prey- and area-specific hunting response (Den Boer 1986). In other situations, opportunistic feeding or deliberate diet mixing could make it much more difficult to distinguish consumption levels and patterns and to quantify the effect of generalist predators on desired biocontrol targets.

Carabid genera such as Amara and Harpalus include a large amount of seeds in their diet (Honek et al. 2007, Talarico et al. 2016), and this has been proposed to regulate the weed seed bank (Bohan et al. 2011a) and reduce weed seedling emergence (White et al. 2007). Granivorous carabids may even be considered agricultural specialists because of their dependence on ruderal plant seeds and their negative response to increased habitat complexity (reduced agricultural habitats; Vanbergen et al. 2010). The role of carabids as weed seed eaters in agriculture is receiving increased attention (Kulkarni et al. 2015). However, estimates of how much carabids contribute to weed seed loss vary greatly, and the activity density of granivorous carabids and weed seed consumption are not always correlated or may be only weakly correlated (Saska 2008, Saska et al. 2008, Davis and Raghu 2010, Petit et al. 2014). This variation can be due to the inherent differences among the studies, such as cropping system design and dominant granivorous species present. However, some of the discrepancies might also stem from differences in carabid foraging and feeding behavior, which, in turn, is influenced by both the spatial environment and species interaction network.

**Figure 1.**
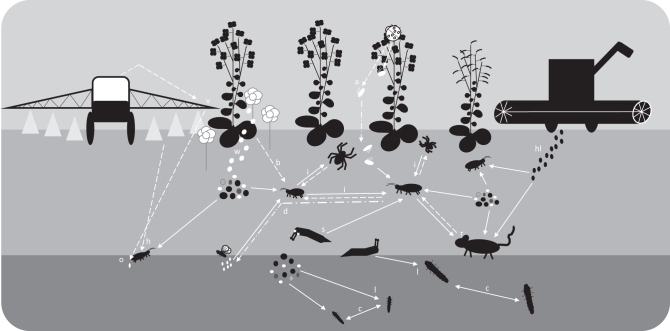
Interaction network of direct and indirect interactions that can affect biocontrol by carabids. The solid lines represent direct (consumptive) interactions. The dashed lines represent indirect (behavioral) interactions. The interactions are explained and referenced in the text; the interactions with a letter are also described here. Herbicides (v) can change the spatial structure and composition of the vegetation. This can have an effect on the availability of weed seeds (w), the behavior (b), and the oviposition (o) choices of carabids. Herbicides can also have direct negative effects on carabid health (h). Intraguild predation (i) and cannibalism (c) can lower biocontrol potential and change carabid behavior. When large carabids eat smaller carabids, they can disrupt biocontrol of pest insect (d), such as flies. Beneficial predators can have additive effects. Aphids (a) that drop to the ground to escape Coccinellidae are vulnerable to predation by carabids. Scavenging behavior (s) can lower carabids contribution to biocontrol. Harvest loss (hl) can increase food availability but could also lower consumption of weed seeds and pest insects. Rodents (r) consume carabids, which can lead to predation risk induced behavioral changes in carabids. Carabid larvae (l) could also contribute to biocontrol by consumption of pest insects and weed seeds.

Carabids are currently being promoted as beneficial insects for both pest reduction and weed seed suppression. However, as Frank and colleagues (2011) indicated, the promotion of carabids as beneficial for both might be conflicting as consumption of one could reduce the consumption of the other, especially for omnivorous species. True omnivory, in which a species feeds on more than one trophic level, can complicate food networks, and carabid biocontrol studies could underestimate carabids’ total contribution if they are focused only on one trophic level. Intraguild predation could also hinder carabids’ ability to provide beneficial services, both between carabids and between carabids and other beneficial predators, such as spiders. Furthermore, nonconsumptive effects of predators can affect the feeding and foraging behavior of carabids. For example, predator cues have been found to increase carabid seed consumption (Blubaugh et al. 2017, Charalabidis et al. 2017), which may reduce the consumption of prey. Therefore, to get a better understanding of the biocontrol contribution of carabids we need to include their foraging and feeding behavior because it is affected by their interaction network. A simplified proposed carabid interaction network in an agricultural setting is displayed in figure 1. Some of the interactions and how they might affect carabid feeding behavior are explained below, and throughout this text we will come back to the interactions in this network and the discussion of how they might affect carabid contribution to biocontrol.

## Foraging and feeding behavior

**Figure 2.**
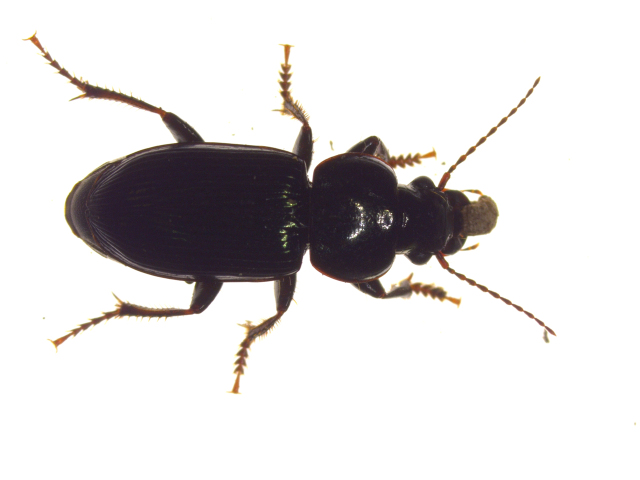
Harpalus amputatus Say carrying a kochia seed.

**Figure 3.**
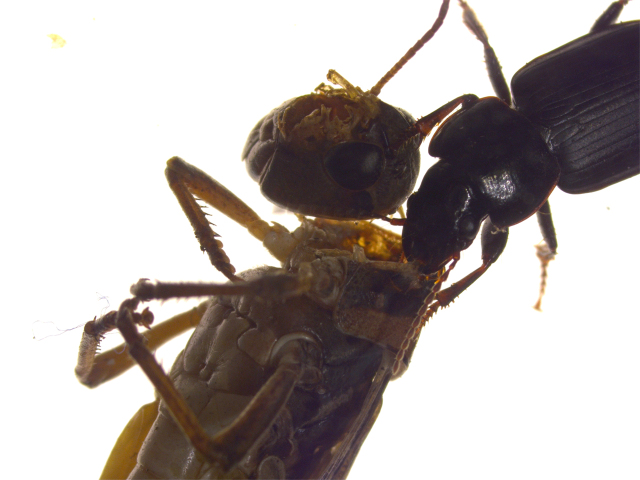
Harpalus amputatus Say scavenging on a grasshopper.

Diet is an important factor in the health of all animals, including carabids. Diet has been found to play a role in the fecundity, larval development, and sexual maturation of carabids (Fawki and Toft 2005, Sasakawa 2009). Both internal factors, such as hunger (Ernsting and Van der Werf 1988), and external factors, such as food density (Dinis et al. 2016), alternative food (Prasad and Snyder 2006), and predation risk (Blubaugh et al. 2017), influence the feeding and foraging behavior of carabids. Similar to various other beneficial insects, many carabids are true omnivores, consuming food from different trophic levels (figures 2 and 3; Larochelle and Lariviere 2003). For example, the consumption of seeds is widespread even among carabid species that are regarded as mainly carnivorous, such as Poecilus cupreus and P. melanarius (Koprdova et al. 2008, Frei et al. 2019). Nonprey foods can be vital parts of the diet of many natural enemies, including for carabids, with regard to reproduction, diapause, and distribution (Lundgren 2009).

The genera Harpalus and Amara are the main groups of granivorous carabids and seeds have been found to be an essential part of the diet of certain species (Klimes and Saska 2010, Harrison and Gallandt 2012). Seeds seem highly important for Amara species, and the inclusion of animal food is generally found to have no benefit or even a negative impact on different Amara life parameters (Fawki and Toft 2005, Saska 2008). However, as Fawki and Toft (2005) noted, Amara species are often found to include animal food in their diet. Therefore, the distinction between granivorous and carnivorous carabids is not clear and might be highly context dependent.

### Alternative food

The availability of food other than the biocontrol target can have a great impact on the outcome of biocontrol efforts. On one hand, alternative food can sustain beneficial predator communities in times when pest populations are low (Eubanks and Denno 1999), and it can mitigate some intraguild predation and cannibalism (Currie et al. 1996, Frank et al. 2010). On the other hand, alternative food can also lower pest predation pressure and, therefore, lower the biocontrol potential of beneficial insects (Symondson et al. 2006). For example, the carabids Calathus granatensis (Vuillifroy) and Pterostichus globosus (Fabricius) were assessed as potential predators on the olive fruit fly Bactrocera oleae Rossi (Diptera: Tephritidae) under laboratory conditions. Although both species ate the olive fruit fly pupae, P. globosus switched to the Mediterranean fruit fly (Ceratitis capitata) when it was more prevalent (Dinis et al. 2016). Therefore, alternative food choices can influence consumption in a density-dependent way. Frank and colleagues (2011) looked at the effect of different alternative food items on carabid recruitment, abundance, and crop protection. Both seeds (Poa pratensis L., Poaceae) and fly pupae (Drosophila melanogaster Meigen, Diptera: Drosophilidae) were found to increase the residence time (determined with mark–recapture experiments) of Harpalus pensylvanicus (DeGeer) and Anisodactylus ovularis (Casey) in the field, but the effect was nonadditive, and the seeds had a greater effect on residence time. They found that the addition of seeds but not fly pupae increased the abundance of mostly omnivorous carabids in the field, and only the addition of seeds led to increased cutworm (Agrotis ipsilon Hufnagel, Lepidoptera: Noctuidae) damage (Frank et al. 2011). Therefore, different alternative food items do not elicit the same effects in carabids. Frank and colleagues’ article, only the addition of seeds diminished crop protection from cutworms by reducing feeding attempts of carabids on cutworm. This study highlights that even though alternative food can increase recruitment and retention of beneficial arthropods, it may not always have the desired effect.

With this in mind, an interesting aspect to consider is the effect of harvest losses on carabid biocontrol potential. Substantial harvest losses of palatable seeds such as canola (Brassica napus L.; Gulden et al. 2003) likely have an effect on carabids in crop fields. Such an abundance of food could increase the attraction and retention of carabids in crop fields. In 2010, outbreaks of Amara spp. in Alberta (Canada) were associated with high canola losses the previous year (Floate and Spence 2015). Conversely, the abundance of canola seed could lower the predation on pest insects or weed seeds (figure 1hl). Although many carabids consume canola seeds (Koprdova et al. 2008), the influence of harvest losses on carabid biocontrol contribution of prey or weed seeds is yet to be explored. Saska (2008) found no correlation between canola consumption and carabid activity density in the field, which suggests that the relation is not straightforward.

### Intraguild predation

Intraguild predation can directly complicate control efforts. For example, wolf spiders (Pardosa littoralis Banks, Aranea: Lycosidae) and mirid bugs (Tytthus vagus Knight, Hemiptera: Miridae) both prey on planthoppers (Prokelisia dolus Osborn, Hemiptera: Delphacidae) and can reduce their populations. However, in habitats in which both predators were present, planthopper populations increased because of intraguild predation of mirid bugs by spiders (Finke and Denno 2003). Attempts to increase biocontrol—for example, by creating beetle banks—can also be disrupted by intraguild predation (Snyder and Wise 1999). Prasad and Snyder (2006) found that beetle banks can indeed increase generalist predators, including carabids, but that this does not necessarily lead to increased biocontrol on desired pest species. In this example, both small egg eating carabids, Staphylinidae (Coleoptera), and the larger P. melanarius benefited from the beetle banks. However, P. melanarius predated heavily on the smaller beetles and rarely on the fly eggs (Diptera), thus lowering the desired biocontrol effect (figure 1d). P. melanarius was also found to disrupt biocontrol of the specialist parasitoid wasp Aphidius ervi Haliday on pea aphids (Acyrthosiphon pisum Harris). P. melanarius predated on the immobilized parasitized aphid mummies, which reduced parasitism and eventually led to an increase in aphid population growth. However, the strength of P. melanarius's effect was dependent on the height of the vegetation and the initial aphid population (Snyder and Ives 2001).

Intraguild predation can also affect granivory, although this is often not included in studies of weed seed predation. One of the few studies to include intraguild predation is Davis and Raghu (2010), who assessed the effects of different biotic and abiotic factors on invertebrate (Carabidae and Gryllidae) weed seed predation. They found spider activity to be negatively correlated with invertebrate seed predation. This suggests that nongranivorous actors can play a significant indirect role on weed seed predation and that it is important to look beyond granivores to understand the drivers of weed seed loss in the field (figure 1i).

Predators can also have additive effects on pest suppression (Snyder 2019). For example, aphids show predator avoidance responses in the presence of lady beetles (Coleoptera: Coccinellidae) by dropping to the ground (Hoki et al. 2014). On the ground, the aphids can fall prey to carabids. Carabids and coccinellids can therefore have an additive effect on aphid suppression, rendering the aphids’ predator avoidance behavior less successful (figure 1a). On the other hand, Prasad and Snyder (2010) found that carabids (multiple species) prefer dropped green peach aphids (Myzus persicae Sulzer) over dipteran eggs and that fly suppression is lowered in the presence of lady beetles (multiple species). This example shows that, in a small four-species network, both direct and indirect interactions are at play, and together, they collectively determine the outcome on lower trophic levels and, therefore, on the biocontrol of specific species.

### Nonconsumptive effects

Although intraguild predation can directly affect biocontrol if beneficial insects eat each other, the presence of predators can also indirectly affect the performance and behavior of other biocontrol species. These indirect effects have been termed behavioral interference, nonconsumptive effects, and trait-mediated effects (Symondson et al. 2002b, Preisser and Bolnick 2008). The many papers produced by the members of the “Does fear matter?” working group led by Preisser and Bolnick (2008) indicated that nonconsumptive predator effects are widespread and are important ecological drivers of trophic cascades (e.g., Preisser et al. 2005, Preisser et al. 2007, Preisser and Bolnick 2008). For example, in a meta-analysis, Preisser and colleagues (2005) found a strong and similar outcome of nonconsumptive and consumptive predator effects and an especially strong effect of nonconsumptive effects on lower tropic levels. Therefore, nonconsumptive effects can cascade and amplify through the food chain. This is supported by another meta-analysis by Preisser and Bolnick (2008), who found a larger effect of predation risk on the prey's foraging behavior than on life-history aspects such as growth, life span, and fecundity. Schmitz and colleagues (2004) even suggested that antipredator behavior is the leading driver of trophic cascades. In carabid studies, nonconsumptive effects are only minimally included and are hardly taken into account when it comes to biocontrol efforts, although these effects could profoundly change the behavior and food choices of carabids and, therefore, their contribution to biocontrol.

The strength of nonconsumptive effects and subsequent effects on lower trophic levels is dependent on a number of factors, such as hunting mode. A meta-analysis by Preisser and colleagues (2007) showed a stronger nonconsumptive effect on prey from a sit-and-pursue strategy than on active predators, although slugs were found to avoid odor cues from the active carabid P. melanarius (Armsworth et al. 2005). Pest compensatory mechanisms also play a role in the outcome of nonconsumptive effects. Tobacco hornworm caterpillars (Manduca sexta L., Lepidoptera: Sphingidae) reduce their feeding in the presence of a predatory stinkbug (Podisus maculiventris Say, Heteroptera: Pentatomidae), and both the consumptive and nonconsumptive effects of the stinkbug reduced plant damage (Thaler and Griffin 2008). However, hornworm caterpillars can compensate for reduced feeding by increasing their assimilation efficiency during predation risk and by compensatory feeding after the risk has subsided (Thaler et al. 2012).

Carabids are the prey items for a variety of vertebrates, such as birds (Vickery et al. 2009), and interfamilial predation is common in the carabid family (Currie et al. 1996). Therefore, it is likely that carabids display behavioral changes because of predation risk by other carabids, as well as other common agricultural animals. Certain predatory carabids, such as P. melanarius, are sometimes used as the predator species in studies on nonconsumptive effects of potential prey. However, examples in which carabids themselves are the study objective of nonconsumptive effects are rarer. Blubaugh and colleagues (2017) observed that increased mice activity led to a reduction in the activity density of carabids in the field, but this did not reduce seed predation. Subsequent laboratory behavioral experiments showed that the carabid Harpalus pensylvanicus DeGeer reduced its activity when exposed to mouse cues, but H. pensylvanicus also increased its seed consumption. Blubaugh and colleagues (2017) hypothesized that this increased seed predation is a predator induced behavioral change in foraging, driven by the fact that seeds are a less risky food item requiring less movement to find (figure 1r). The preference and acceptance for certain seeds can also be affected by perceived predation risk. The granivorous Harpalus affinis Schrank accepted intermediate preferred dandelion seeds (Taraxacum officinale Wigg) quicker and ate more seeds when exposed to chemical cues from P. melanarius (Charalabidis et al. 2017). As these examples indicate and as Charalabidis and colleagues (2017) also suggested, weed seed predation is not determined just by the community of granivores but, rather, by the entire interaction network they are part of.

## The spatial structure of an interaction network

The spatial structure of agroecosystems also has an effect on the strength of the interactions between the animals living in it. Increased habitat diversity and structural habitat complexity have been argued to support higher (beneficial) arthropod diversity and ecosystem services in agricultural landscapes (Langellotto and Denno 2004, Landis 2017, Lichtenberg et al. 2017). However, this cannot simply be generalized and extrapolated across all (potentially) beneficial arthropod groups and services and landscape scales. Vanbergen and colleagues (2005) reported an increase in carabid diversity and abundance along a gradient from sites dominated by forest to sites dominated by agricultural land (at a scale of 1 square kilometer). Intermediate sites, with the highest habitat diversity and intermediate disturbance did not support the highest carabid diversity or activity density, contrary to their expectation. The suitability of carabids to disturbed man-made environments, such as crop fields, is exemplified by the fact that many reproduce and overwinter within the soil of cropped fields (Noordhuis et al. 2001). Noncrop habitats, such as hedge rows, can even limit the dispersal of carabids (Thomas et al. 1998). Moreover, predation on carabids themselves has been found to increase with increased habitat complexity (at a scale of 72 hectares; Birthisel et al. 2014). However, what is deemed a simple or a complex habitat is ambiguous; one simple habitat might support more beneficial arthropods than another simple habitat.

Habitat complexity and diversity in relation to arthropods in agroecosystems are often discussed on a relatively large scale (landscape level). However, interactions of individual arthropods can be affected by a much smaller spatial structure (field or even plant level). Different crop fields support different spatial structures (e.g., different crop architecture, amount of weeds, and amount of litter), which means that arthropods may contribute differently to biocontrol depending both on their location within the field and the location of the field in the larger area. For example, the earlier mentioned antagonistic relation between mirids and wolf spiders diminishes in complex vegetation, which strengthens the suppression of their shared prey, planthoppers (Finke and Denno 2002). Although structural complexity can reduce intraguild predation, it can also lower predation pressure on prey. Birkhofer and colleagues (2011) found similar activity density but different aggregations of carabids, spiders, and collembolan between conventional and organic wheat fields. These differences were related to structural differences in the crop (the organic wheat was taller). They argued that the intraguild interactions (spider–carabid) were reduced in the more complex organic wheat. However, the organic wheat also provided more refuge for the collembolan, which resulted in a higher coexistence of predators and prey. Whether the lower coexistence in simple habitats was due to increased predation or to predator avoidance behavior was not investigated. It is not known whether increased structural complexity in crop fields will aid carabid biocontrol of pest species, and this will likely depend on the pest species and the strength of intraguild predation. It will be interesting to see this further explored.

The spatial habitat can also affect weed seed predation, largely because the structure of the vegetation can affect the type of seed predators. Orrock and colleagues (2003) found that invertebrates removed more seeds from patches in an open environment, whereas rodents removed more seeds in patches connected with vegetative corridors. Although the total seed consumption remained equal among the patches, the identity of the granivores changed, and with it, the interaction network. Jonason and colleagues (2013) observed higher weed seed predation in simple landscapes positively correlated to carabid species richness, regardless of whether the field was organic or conventionally farmed. On a smaller scale, Cromar and colleagues (1999) reported an effect of crop residue on seed predation in corn fields in which carabids were the dominant granivores. They found higher average weed seed predation in fields with corn residue than in those with soybean and wheat residue. Vegetation cover in crop fields has also been found to have a positive effect on the weed seed predation of both vertebrates and invertebrates (mostly carabidae; Meiss et al. 2010) and of carabids specifically (Blubaugh et al. 2016). Blubaugh and colleagues (2016) reported strong effects of cover crops on carabid activity density and seed predation, as was confirmed by gut content analyses. Meisse and colleagues (2010) indicated that the positive effect of vegetation cover can be due to a number of nonmutually exclusive causes, such as creating favorable microclimates, substrate for reproduction, alternative food sources, and lower predation risk. How crop field architecture can be designed to optimize biocontrol could be an interesting avenue of agronomic study.

## The effect of pesticides on carabid network interactions

Agrochemicals (herbicides, insecticides, fungicides, and synthetic fertilizers) can have direct negative effects on insect health (figure 1h), and they can change the spatial structure (figure 1v) and food availability of agricultural fields (figure 1w), which can change insect interactions. There are many examples of the negative effects of agrochemicals on nontarget carabids (Kunkel et al. 2001, Mauchline et al. 2004, Giglio et al. 2011, Cavaliere et al. 2019), how they can change carabid community structure (Nash et al. 2008), and how agroecosystems with reduced chemical input can support higher carabid abundances (Navntoft et al. 2006). Because insecticides can both kill and induce sublethal changes in carabids and because these effects are not symmetric across species, they can change carabid interaction networks. For example, reduced application of insecticides and herbicides in winter wheat was found to increase total carabid abundance, but it reduced the genera Bembidion, Synuchus, and Trechus (Navntoft et al. 2006). The reduction of these three genera could have been the result of vegetation differences due to reduced herbicide use or the result of competitive changes by larger predatory carabids. If carabids with certain feeding habits are more heavily affected by pesticide use than others, then the carabid community can change, and, with it, so can their biocontrol potential.

Insecticide application can alter carabid biocontrol even if it has no direct negative effects on the beetles themselves. P. melanarius has been found to prefer dead or immobile prey over live prey (Ferrante et al. 2017). Insecticide application can be followed by a period of increased abundance of dead or immobile prey and if carabids feed preferentially on these prey they likely fulfill no additive biocontrol service (figure 1s). In addition, this contaminated prey can be an (additional) harmful source of insecticide exposure. If carabids avoid insecticide killed prey this could be mitigated, but there are several examples to the contrary (Kunkel et al. 2001, Langan et al. 2001, Mauchline et al. 2004). To illustrate, P. melanarius, Pterostichus madidus (Fabricius), and Nebria brevicollis (Fabricius) were shown to suffer great mortality when fed on 10%, 25%, and 100% (of field application) dimethoate dosed aphid prey (Sitobion avenae Fabricius). Nevertheless, these beetles made no distinction between treated and untreated dead prey in a choice test (Mauchline et al. 2004).

Another way in which carabids may be exposed to insecticides is via transgenic insecticidal crops. Their exposure can be direct, by eating plant parts or by contact with root exudates, or can be indirect, via the consumption of herbivores that fed on the transgenic crops (Peterson et al. 2009). Bacillus thuringiensis (Bt) toxin-expressing crops are the main transgenic insecticidal crops, and Bt toxins have been found in the gut content of multiple carabid species from fields with Bt crops and Bt crop residue (Zwahlen and Andow 2005, Peterson et al. 2009). However, adult carabids fed prey that were reared on transgenic insecticidal crops did not necessarily experience negative reductions in survival, weight gain, or reproduction (Ferry et al. 2006, Mulligan et al. 2006).

These effects should not be generalized across different Bt toxins and beneficial insect groups. For example, Stephens and colleagues (2012) found only subtle effects of Bt maize on carabids but reported strong negative effects on Harmonia axyridus Pallas (Coleoptera: Coccinellid) that consumed aphids that had fed on Bt maize. Furthermore, transgenic insecticidal crops may induce behavioral changes in arthropods, such as avoidance of prey on these crops because of reduced prey quality (Ferry et al. 2006). Nevertheless, Han and colleagues (2016) concluded that the overall effects of transgenic insecticidal crops on the behavior of natural enemies is limited. However, alternative pesticides exposure routes, such as transgenic crops and seed treatments, and their indirect effects on prey quality or quantity should not be overlooked when assessing pesticide impact on beneficial arthropods.

## Connected carabids, looking forward

We think it is now well established that carabids are a common part of many agroecosystems with the potential to significantly contribute to the reduction of pests and weeds. However, what is not yet well understood is their place in agroecosystem interaction networks and how both direct and indirect interactions influence their contribution to biocontrol. We think that in order to elucidate this, research on carabid biocontrol needs to move away from a relatively simple approach focused on carabid consumption of one pest or a group of pests and toward a more ecological approach in which the network of interactions among pests, carabids, and other organisms within its spatial structure is evaluated, with equal focus on both direct and indirect interactions.

Studying the network of agroinvertebrates can bring to light different effects of agronomic practices as opposed to more traditional species-oriented ecological approaches (Ma et al. 2019). Although agroecosystems might be simplified systems, that does not mean they support simple networks. A recent overview article from Vandermeer and colleagues (2019), exemplified the kind of complex interactions agroecosystems can support. In coffee plantations, various species of ants, flies, green coffee scales, coffee berry borers, fungal pathogens, and a predatory beetle have been shown to interact in direct and indirect ways, shaping each other's populations and spatial distributions, with the same species providing both beneficial and harmful services, depending on the dominant players in the network (Vandermeer et al. 2019).

Studying the community of arthropods in an agroecosystem can be a daunting task, because the number of actors in community networks can be vast, and a priori knowledge of the most important players is not always available. However, there are tools available that can aid in this endeavor and that can show direct (consumptive) interactions. Modern tools, such as machine learning, modeling, and next-generation sequencing, can be used to direct and aid in community network analyses (Vacher et al. 2016). For example, Bohan and colleagues (2011b) used logic-based machine learning to generate a hypothetical food network on the basis of invertebrate field data. The network confirmed a number of known interactions but also hypothesized unknown interactions. Furthermore, it indicated a more important role of carabid larvae than the authors had previously thought (figure 1l). These discoveries could be interesting avenues for further research and can be used to both generate and prioritize interaction network hypotheses.

Modeling can also be used to assess the most useful targets for biocontrol efforts. Kean and colleagues (2003) used a modeling approach to assess how a conservation biocontrol effort (supplementation of floral resources) affects different aspects of natural enemy biology and subsequent effects on prey densities. They concluded that the reproductive rate of natural enemies such as parasitoids is more important for biocontrol than, for example, their longevity.

Vacher and colleagues (2016) argued that next-generation sequencing could be used to obtain information of the species and potential links in a network from environmental DNA (from, e.g., soil, feces, or gut content samples) and that this, in combination with modeling and machine learning, can be used to build interaction networks. Vacher and colleagues (2016) used next-generation sequencing to evaluate the relative contribution of carabids to pest control and intraguild predation on the basis of gut content analyses in 503 specimens from 14 species. They found their biocontrol service and harmful impact to be about equal. Novel statistical approaches can also be very insightful when it comes to identifying key biocontrol species or groups. Carbonne and colleagues (2019) used a regression tree analysis to identify key carabid taxa and combinations responsible for Viola arvensis Murray seed consumption. This approach proved useful because it consistently identified the key seed consuming species. Furthermore, they found that intraguild predation played a minor role in V. arvensis seed consumption and that, in many fields, seed predation was limited because of low abundance of key species. Together, these techniques can be useful tools to advance the study of potentially beneficial insects, such as carabids.

There is a whole web of indirect interactions among carabids and other animals in agroecosystems that we have yet to explore. Building on the work by Blubaugh and colleagues (2017) and Charalabidis and colleagues (2017), starting with a two-species interaction network, carabid behavior can be studied in increasingly complex (realistic) interaction networks. As different players, food choices, and spatial settings are included in such studies, carabid biocontrol potential can be evaluated within its appropriate context. The modeling of interaction networks can be used as a starting point for picking the players for such studies. In contrast, once more is known about how both consumptive and nonconsumptive effects can affect a pest, this can be related back to data on field communities and their level of pest control.

Another way in which arthropod interactions can be assessed is via camera traps and neural network learning. Hansen and colleagues (2020) had a 74.9% and 51.9% success rate with such a technique in identifying carabids from museum specimen images to genus and species, respectively. Because the accuracy of these techniques is only likely to increase as more and more training data sets are fed into these networks, this can become a very valuable technique for future fieldwork. Camera traps can replace destructive sampling techniques, such as pitfall traps, with the added advantage that they will not only sample which arthropods are active in an area but also when. Camera traps can give insight in potential additive or disruptive indirect interactions among different beneficial arthropod groups that cannot be obtained from pitfall traps, which are left in the field for days to weeks.

Although they are interesting in its own right, greater understanding of agroecosystem interaction networks would hopefully lead to more sustainable agricultural systems that fully exploit natural weed seed and pest reduction by beneficial arthropods, such as carabids. Although the direct effect of beneficial insects may be obvious, indirect effects can also be exploited for pest control and crop protection. For example, Rypstra and Buddle (2012) showed that spider silk reduced herbivory by both Japanese beetles (Popillia japonica Newman, Coleoptera: Scarabaeidae) and Mexican bean beetles (Epilachna varivestis Mulsant, Coleoptera: Coccinellidae) in lab and semifield trails. Blaustein and colleagues (2004) suggested that predator cues that deter mosquitos from oviposition in pools could be used to make more environmentally friendly chemical mosquito control. For an overview of other behavior-based insect control options, such as mating disruption, see Allan (2018). Exploitation of predator cue induced behavior changes in pests could be a great additional tool for biocontrol strategies, without the negative side effects agrochemicals can have on nontarget beneficial predators. But, as Allan (2018) indicated, behavior based biocontrol strategies could also lose their proficiency because of habituation, behavior adaptation, and resistance.

Studying these interaction networks occurs in ever-changing environments. Agricultural landscapes and agronomic practices change as new techniques are adopted and new societal values are acted on. Furthermore, other changes such as climate change, increase in artificial night light (Owens and Lewis 2018), invasive species, carbon dioxide level elevations, and nitrogen deposition (to name a few), can also influence agroecosystem communities. This has already been shown in practice; the carabid ­species diversity decline in a nature reserve in Germany has been associated with climate change, with spring breeding species being most negatively affect as their larvae face warmer and dryer summers (Homburg et al. 2019). The introduction of new species such as, P. melanarius (Niemelä and Spence 1999) and Nebria brevicollis Fabricius (LaBonte 2011) in North America may not only change the players in an interaction network but also the presence or strength of their interactions. P. melanarius has been shown to change community network interactions and disrupt biocontrol efforts (Prasad and Snyder 2006). New species interactions can also emerge because of range expansion driven by climate change. Rising temperatures can lead to insect ranges shifting and expanding (Musolin 2007), which can lead to novel interactions, especially because change in insect ranges is not expected to be symmetrical across species (Berg et al. 2010).

## Conclusions

In conclusion, biocontrol services provided by carabids are highly promising but quite variable. Because the foraging and feeding behavior of this omnivorous group of insects is greatly influenced by their interaction network and spatial environment, predicting and using carabid biocontrol services has proven difficult. In the present article, we have highlighted some of the many ways that interactions with other animals in the agroecosystem may affect natural biocontrol contributions from carabids. We highly encourage more work on the effects of interaction networks on carabids and other beneficial insects and their biocontrol services. Further research on the mechanisms that underlie these relationships should also be explored. We believe such work can lay the groundwork for designing more sustainable agricultural productions systems, especially with regard to long-term weed and pest management.

## Author Biographical

Stefanie E. de Heij (sedeheij@gmail.com) and Christian J. Willenborg are affiliated with the Department of Agriculture at the University of Saskatchewan, in Saskatoon, Saskatchewan, Canada.
